# Preparation, Characterization, Stability and *In Vitro* Release of a Pea Protein Fibril-Based Iron Fortificant via Self-Assembly

**DOI:** 10.3390/foods13223558

**Published:** 2024-11-07

**Authors:** Xiaoting Chen, Jiang Yi, Zhen Wen, Yuting Fan

**Affiliations:** 1 Shenzhen Key Laboratory of Food Macromolecules Science and Processing, College of Chemistry and Environmental Engineering, Shenzhen University, Shenzhen 518060, China; yijiangjnu@gmail.com (X.C.); wenzhen@szu.edu.cn (Z.W.); 2 School of Public Health, Shenzhen University Medical School, Shenzhen University, Shenzhen 518060, China

**Keywords:** pea protein, amyloid fibrils, iron fortifier, reducing effect

## Abstract

It is assumed that the stability and bioaccessibility of iron ions in iron–pea protein fibril (Fe-Fib PP) nanocomposite can be remarkably enhanced, and Fe-Fib PP exhibits great potential as an effective iron fortificant. Fe-Fib PP, a stable and effective iron supplement, was fabricated based on the reducing property of pea protein fibrils, derived from pea protein through thermal treatment at pH 2.0. The results demonstrated that the reducing power of iron was remarkably affected by fibril concentration and fibrillization degree. The reducing power of pea protein fibrils gradually enhanced from 0.31 to 0.92 with the increase in incubation time from 0 to 48 h. Compared with iron nanoparticles (Fe–Nano), Fe-Fib PP possessed much higher dispersibility. Additionally, the stability of iron in Fe-Fib PP was significantly higher than that in Fe–Nano under different storage conditions. X-ray photoelectron spectroscopy (XPS) outcomes revealed Fe (II) content in Fe-Fib PP (70.75 ± 0.65%) was remarkably higher than that of Fe–Nano (56.05 ± 0.50%). In addition, the bioaccessibility of Fe (II) dramatically improved from 42.7% to 62.8% using PP fibrils as carriers. The findings suggest that Fe-Fib PP is an effective iron nutrition enhancer.

## 1. Introduction

Anemia is medically related to the decrease in red blood cells, which mainly manifests as insufficient oxygen-carrying capacity and the inability to meet the demand for oxygen supply, thus affecting human physiological activities [1]. When the total iron intake is not enough, or the absorption rate of iron is low, the concentration of heme in the blood is low, resulting in iron deficiency anemia. Additionally, the proportion of pregnant women and preschool children is large in the iron deficiency anemia (IDA) group. At present, approximately a quarter of the global population is affected by this condition, and iron deficiency is the main cause of anemia [2]. As an essential trace element for human beings, iron plays an important role in transporting oxygen, enhancing metabolism, regulating immunity, and enhancing nucleic acid synthesis and repair [3]. Iron deficiency anemia can lead to weight loss and frequent intestinal and respiratory infections in children, primarily due to an impaired T-lymphocyte immune response with the occurrence of iron deficiency [4]. In order to effectively alleviate the effects of iron deficiency anemia (IDA), the World Health Organization (WHO) has adopted a strategy of dietary iron fortification to enhance iron absorption through food fortifications. Generally, the iron fortification of food is an effective, sustainable, and low-cost approach for reducing iron deficiency anemia (IDA).

However, the chemical form of iron and the concentration of iron in food iron fortificants often cause undesirable sensory changes and mitigate food acceptability and bioavailability. Traditional iron fortifications also exhibit low water solubility [5]. For example, the use of polyamino acid complex comprising iron and ferrous glycinate as food iron fortifiers often causes undesirable sensory changes and intestinal side effects that lower the acceptability and availability of iron fortifiers [6]. Therefore, the main challenge for food iron fortification is to increase iron absorption. Iron generally exists in two valence states: reduced ferrous (Fe (II)) and iron oxide (Fe (III)). Only Fe (II) can be effectively absorbed by duodenal epithelial cells, and Fe (III) must first be reduced to Fe (II) by reducing agents or reductase in intestinal epithelial cells before absorption. Compared with trivalent iron (Fe (III)), bivalent iron (Fe (II)) is more conducive to absorption by the human body [7]. In the past few decades, food scientists have put plenty of attention on some iron nanoparticles as food fortifiers to replace the commonly used iron salt due to their excellent bioavailability and low side effects. Nevertheless, iron nanoparticles are prone to oxidation and exhibit poor colloidal stability. Recently, iron nanoparticle composites have been applied as iron-fortifying agents. Shen et al. used β-lactoglobulin fibrils as effective carriers to prepare a stable protein fibril–iron nanoparticle composite material as an iron nutrition enhancer in food [8]. Whey protein isolate fibrils possess high antioxidant activity, and iron-loaded whey protein isolate fibrils exhibit relatively high stability. Xiang et al. studied the reduction effect of soy protein fibrils on iron ions and the effect of fibril concentration on iron reduction [9]. However, the bioaccessibility of iron ions on soy protein fibrils has not been investigated [9]. Furthermore, allergy to soy proteins or whey proteins is detrimental to the application of iron ion-loaded soy protein nanofibrils in food systems.

Food protein fibrils also have the capacity to form a winding network structure. The structural unit polypeptide in protein fibril structure has a high aspect ratio, which is conducive to enhancing the reducibility. Therefore, it can be used as a great carrier of bioactive materials [10]. In the past, animal proteins were usually used in food production and functional food applications. Plant proteins are far more crucial considering sustainability, richness, and cost [11]. Pea protein (PP) mainly consists of four major classes (globulin, albumin, prolamin, and glutelin) storage proteins [12]. PP is a cheap, abundant, and environmentally friendly pulse protein, which may be utilized as a suitable and efficient alternative to soybean or milk proteins in nutraceutical delivery systems [13]. Its fibrils can be formed by heating at pH far away from the isoelectric point [14]. During the heating process, PP hydrolyzes and then self-assembles into fibrils, forming mature fibrils after about 20 h incubation [14]. It has been shown that the fibrillated PP has markedly higher antioxidant and free radical scavenging activity *in vitro* than the original form due to the production of bioactive peptides, which can maintain the stability and activity of encapsulated nutrients [15]. Furthermore, the network structure and the high polypeptide aspect ratio endow its good stability and load capacity, making it an ideal carrier material for bioactive nutrients [16].

There are some arguments to deduce that PP fibrils may possess the properties of reducing iron ions and can be used as a great carrier of iron enhancement for food iron nanoparticles [17]. In this study, an iron nanoparticle composited with PP fibrils as the carrier was fabricated, and the reduction effect of PP fibrils on iron (III) and iron nanoparticles was thoroughly studied. The influences of different fibrillation degrees and fibril concentrations on iron reduction capacity were compared. Additionally, the storage stability of the PP fibril–iron nanoparticle composite under different storage conditions was fully evaluated. The bioaccessibility of Fe (II) in PP-based fibril nanocomposites during *in vitro* simulated gastrointestinal digestion was also studied. The goal of this research was to develop and obtain PP-based fibrils as efficient carriers for iron fortification to explore a new effective way to prevent and solve IDA.

## 2. Materials and Methods

### 2.1. Materials

Pea protein isolate (PPI, protein content 85%) was freely supplied by Roquette (Lestrem, France). Thioflavin T (ThT), FeCl_3_, TEMED, SDS, Tris-HCl (pH 6.7), Tris-HCl (pH 8.9), and HCl were purchased from Macklin Biochemical Technology (Shanghai, China). Sample buffer and marker for SDS-PAGE were purchased from Yuanye Biotechnology (Shanghai, China). Sodium borohydride (NaBH_4_) and 1, 10-phenanthroline were purchased from Aladdin (Shanghai, China). All chemicals used were at least of reagent grade. Deionized water was used in all experiments.

### 2.2. PP Fibril Preparation

PP fibrils were prepared according to the method published in our group [15], with minor modifications. The PP powder was dissolved at 40 g/L, stirred for 4 h, refrigerated at 4 °C, and hydrated overnight. The pH was set to 2.0 with 6 mol/L hydrochloric acid, centrifuged (10,000× *g*, 20 min, 4 °C), filtered to remove the undissolved protein, and then heated in a water bath (SCG-4, Scientz, Ningbo, China) at 85 °C. The incubation time was 0, 1, 2, 4, 8, 12, 24, and 48 h, respectively. After heating, the solution of PP fiber was cooled in an ice bath and stored in a refrigerator at 4 °C or lyophilized for further analysis.

### 2.3. Preparation of Iron–Pea Protein Fibril (Fe-Fib PP) Nanocomposites

Fe-Fib PP nanocomposites were prepared according to a recently reported method [9], with appropriate modifications. Mature PP fibrils (10 g/L, incubation for 24 h) were fully dissolved in deionized water (pH 2.0), and a 0.1 mol/L FeCl_3_ solution (deionized water, pH 2.0) was added. The final mass ratio of Fe: Fib PP was set at 1:8 (*w*/*w*). The mixture was stirred at 300 rpm and incubated for 30 min at room temperature (25 °C). After that, 0.1 mol/L NaBH_4_ solution was slowly added and stirred, followed by incubation for 2 h to form Fe-Fib PP nanocomposites. The final NaBH_4_/FeCl_3_ molar ratio was 1:1. Iron nanoparticles (Fe–Nano) were fabricated according to the method above without PP fibrils.

### 2.4. Thioflavin T (ThT) Fluorescence Analysis

The formation of PP fibrils and the kinetics can be analyzed with a fluorescent ThT probe since it can effectively bind with the β-sheet of protein fibrils. The determination of ThT fluorescence was performed based on the previously described method [18]. The ThT fluorescence spectra of PP fibrils incubated at 85 °C (pH 2.0) for various times (0, 1, 2, 4, 8, 12, 24, and 48 h) were recorded with a fluorescence spectrophotometer (F-7100, Hitachi, Tokyo, Japan). First, 100 mL of ThT stock solution (0.8 g/L) was prepared using 10 mmol/L phosphate buffer (PB, pH 7.0) with 150 mmol/L NaCl. Then, the ThT working solution was obtained by dilution 50 times with the same PB. Before detection, 200 μL of each PP fibril solution was mixed with 2 mL of working solution. The excitation wavelength was 400 nm, and the emission wavelength range was between 410 and 600 nm. The ThT fluorescence intensity of the working solution was used as the background value.

### 2.5. SDS-PAGE

The molecular weight (Mw) distribution of PP during fibrillation for various heating times (1, 2, 4, 6, 8, 12, and 24 h) was studied with SDS-PAGE in the presence of β-mercaptoethanol. The gel was composed of 15% separation gel and 5% packing gel. Briefly, 10 μL of each sample (20 μg protein in total) was loaded into each well, and the experiment was performed with 120 V in the Laemmli system. Afterward, the gel was stained with 0.25% a Coomassie Brilliant Blue R-250 solution, and ethanol–acetic acid–distilled water (50:75:875, *v*/*v*/*v*) solution was used to decolorize the gel.

### 2.6. Atomic Force Microscopy (AFM)

About 2 μL of PP fibril solution (1 mg/L, pH 2.0) was placed on a mica sheet and evenly spread with a pipette tip. The mica sheet was then stored on a Petri dish and dried with a dryer (300 mm, Xiangbo, Changsha, China) for 24 h. AFM images were taken with an atomic force microscope (Multimode 8, Bruker, Karlsruhe, Germany), and NanoScope Analysis 1.5 software was used for the processing. For air imaging, an uncoated silicon cantilever beam (RTESP type, Olympus type, Veeco type, OMCL-AC type) (Bruker, Karlsruhe, Germany)) was used. The driving frequency was about 300 kHz, and the scanning frequency was between 0.3 and 0.8 Hz [19].

### 2.7. Free Sulfhydryl Content

The content of the free sulfhydryl group was determined based on a classic protocol [20], with some adjustments. A Tri-Gly buffer solution (1 L) was prepared using 10.4 g of trimethylol aminomethane, 6.9 g of glycine, and 1.2 g of ethylenediamine tetraacetic acid, and the final pH was adjusted to 8.0. The working solution was prepared by dispersing 5,5′-dithiobis(2-nitrobenzoic acid) in the Tris-Gly buffer solution to a final concentration of 4 mg/mL. The urea solution (8 M) was prepared with the Tris-Gly buffer solution (urea reagent). After that, a sample solution (0.5 mL) was thoroughly mixed with 2.5 mL of urea reagent and 20 μL of the working solution, and the reaction was carried out at 25 °C for 1 h. The UV absorption value was then determined at 412 nm. Finally, the following formula was used to calculate the free sulfhydryl content:

(1) $$\mathrm{SH}\;(µ\mathrm{mol}/g)=\frac{73.53\times A_{412}\times D}{C}$$

where *D* is the dilution factor, and *C* is the concentration of the sample.

### 2.8. Antioxidant Activity

#### 2.8.1. Reducing Power

The reducing power of PP fibrils as a function of fibrillation degree was detected according to a method described previously [21]. In brief, 0.5 mL of protein solution (10 g/L) was mixed with 0.5 mL of PB (0.2 mol/L, pH 6.6) and 0.5 mL of potassium ferricyanide (10 g/L). The mixture was then incubated at 50 °C for 20 min. After that, 0.5 mL of trichloroacetic acid (TCA, 10%, *w*/*v*) was added, and the supernatant was obtained by centrifugation (1500× *g*, 10 min). Additionally, 1 mL of distilled water and 1 mL of FeCl_3_ were added to 1 mL of supernatant and mixed. Finally, the absorbance was measured at 700 nm after incubation at room temperature for 10 min.

#### 2.8.2. ABTS^+^ Scavenging Activity

ABTS^+^ scavenging ability measurement was performed referring to the protocol described previously [22]. Briefly, 20 μL of the diluted sample solution (5 mg/mL protein) was mixed with 4 mL of ABTS^+^ diluted working solution and placed in the dark for 6 min. The ABTS^+^ diluted working solution at a concentration with an absorbance of 0.70 (±0.02) at 734 nm was prepared and used. Absorbance was then measured at 734 nm. The equation below was used to calculate the ABTS^+^ scavenging activity.

ABTS^+^ scavenging ability (%) = [1 − (sample/blank)] × 100 (2)

#### 2.8.3. Reducing Effect on Iron Ions by PP Fibrils

In order to evaluate the reducing ability of PP fibrils with different degrees of fibrillation to Fe (III) ion, the Fe (II) ion content in PP fibrils was determined using a phenanthroline colorimetry approach [23]. Briefly, 200 µL of PP fibril solution (10 g/L) was mixed with 0.2 mL and 0.1 mol/L of FeCl_3_⋅6H_2_O solution, and 1 mL of 1,10-phenanthroline (5 mmol/L) was added. An appropriate amount of FeCl_3_⋅6H_2_O solution and an excessive amount of 1,10-phenanthroline solution were added to ensure the complete reaction of amino acids with reducing capacity in the protein fibrils with Fe (III) ions. An orange–red complex was formed when Fe (II) interacted with 1,10-phenanthroline, and the absorbance was measured at 512 nm using a microplate reader (SYNEEGY H1, BioTek, Winooski, VT, USA). The absorbance changes during the total 25 h reaction were recorded at an interval of 15 min. FeCl_3_ solution was used as a blank control to exclude the influence of the reaction system itself.

### 2.9. Transmission Electron Microscopy (TEM)

The micromorphology and size of Fe-Fib PP and Fib PP were observed using a transmission electron microscope (JEM−2100, JEOL Co., Tokyo, Japan) at 80 kV. Firstly, Fe-Fib PP and Fib PP were diluted with deionized water (pH 2.0) to reach a final protein concentration of 0.05% (*w*/*v*). Approximately 10 μL of the solution was then put on the copper net with a carbon supporting film, kept for 3 min, and dried in the oven at 40 °C for 24 h before observation.

### 2.10. Stability Evaluation

The heat stability of Fe-Fib PP nanocomposites during 24 h storage at varied temperatures (25, 50, and 80 °C) was evaluated as a function of Fe (II) content. The storage stability of Fe-Fib PP nanocomposites during 30 d storage at room temperature exposed to light was evaluated as a function of Fe (II) content. Fe–Nano was used as control.

### 2.11. X-Ray Photoelectron Spectroscopy (XPS)

Both Fe–Nano and Fe-Fib-PP solutions were freeze-dried to obtain solid powders, and their XPS spectra were determined with a K-Alpha X-ray photoelectron spectrometer (Thermo Fisher, Waltham, MA, USA). The X-ray source was an Alka micro concentrated monochromator, and the energy of the ion source was 1486.6 eV. The vacuum of the system was up to 10^−7^ PA. The XPS ion-depth analysis experiments were all carried out with a differential pumping ion gun (IQE-12/38, Thermo Fisher, Waltham, MA, USA) using argon ion beam etching technology, with a working voltage of 100–4000 eV. All binding energy data were calibrated at a C1s peak of 284.6eV. XPSPEAK 41 software was used to process spectra, and Origin 2021 software was used to draw the XPS energy spectrum of the valence distribution of iron elements.

### 2.12. Bioaccessibility

The *in vitro* bioaccessibility of Fe (II) in Fe-Fib PP was studied with a simulated gastrointestinal tract according to a previously described protocol [24]. The simulated gastric fluid (SGF, pH 2.0) was composed of 2 mg/mL NaCl, 7 mL/L HCl, and 3.2 mg/mL pepsin, and simulated intestinal fluid (SIF, pH 7.0) consisting of 6.8 mg/mL KH_2_PO_4_ and 1% (*w*/*v*) pancreatin. In brief, 10 mL of Fe–Nano and Fe-Fib PP solution (concentration of 10 mg/mL) was mixed with 10 mL of simulated gastric juice, and the pH value was adjusted to 2.0 with HCl. Gastric digestion was performed for 2 h at 250 rpm at 37 °C. Then, 10 mL of the digesta was added to 10 mL of simulated intestinal fluid, the pH was adjusted to 7.0 immediately with NaOH, and intestinal digestion was performed for 2 h at 250 rpm at 37 °C. After digestion, aliquots of digesta were collected, and the percentage of Fe (II) release was detected by phenanthroline colorimetry at room temperature for 5 min.

Fe (II) bioaccessibility (%) = Fe (II) release amount/total amount of Fe (II) × 100(3)

### 2.13. Statistical Analysis

The experiments were carried out in triplicate, and the results are reported as means ± STD. Statistical significance (*p* < 0.05) between the mean values was determined with variance analysis (ANOVA) and Duncan’s test using SPSS Statistics (SPSS 23.0, IBM, Chicago, IL, USA).

## 3. Results and Discussion

### 3.1. Characteristics of Pea Protein Fibrils

#### 3.1.1. ThT Fluorescence

**Figure 1:**
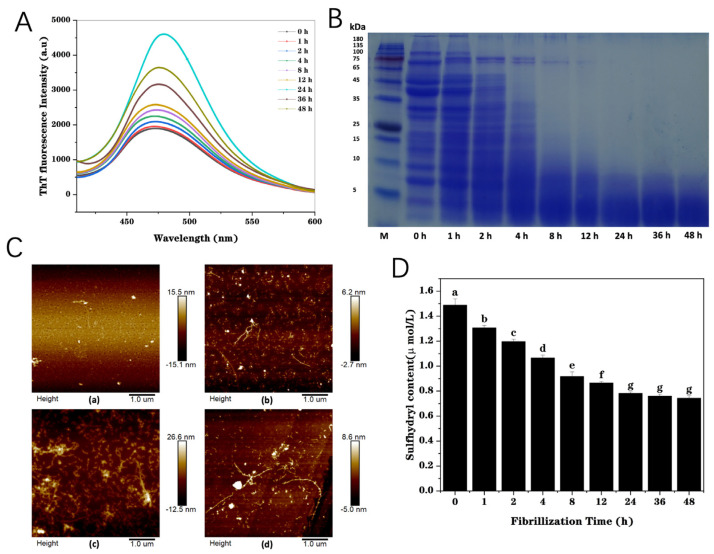
Thioflavin T (ThT) fluorescence spectra of pea protein heated at 85 °C for various times (0, 1, 2, 4, 8, 12, 24, 36, and 48 h) at pH 2.0 (**A**). SDS-PAGE profiles of pea protein during fibrillation (0, 1, 2, 4, 8, 12, 24, 36, and 48 h) (**B**). AFM images of pea protein heated at 85 °C for different times ((a–d) are samples heated for 4, 12, 24, and 36 h, respectively) at pH 2.0 (**C**). Free sulfhydryl content of pea protein heating at 85 °C for different times (0, 1, 2, 4, 8, 12, 24, 36, and 48 h) at pH 2.0 (**D**). Various letters (a–g) mean the significant difference (*p* < 0.05).

Generally, protein fibrils dominantly feature the formation of a large number of β-sheet structures [25]. The formation of protein amyloid fibrils can be sensitively detected with the fluorescent ThT [18]. The higher the fluorescence intensity is, the more the amount of β-sheet structure, demonstrating the assembly of the protein fibrils [26]. As depicted in Figure 1A, the fluorescence intensity of PP peaked at 475 nm and gradually increased with the increase in heating time, indicating that the amount of β-sheet structure increased, which confirmed the formation of fibril structure. A slight increase in the fluorescence was observed during 0–12 h heating, but there was an obvious soaring during 12–24 h heating. A maximum fluorescence intensity value was observed at 24 h heating, demonstrating the formation of β-sheet structure. There was a decreasing trend when further increasing the thermal treatment time from 24 to 48 h, suggesting that long-time acid-heat treatments could lead to the partial destruction of highly ordered structures [27]. It is preliminarily concluded that the formation of fibrils is initiated by the hydrolyzation of protein molecules into polypeptides, followed by self-assembly into protein fibrils, and the hydrolysis and self-assembly occurred simultaneously. The outcomes demonstrated that mature PP fibrils could be formed after 24 h heating at pH 2.0.

#### 3.1.2. SDS-PAGE

The relative molecular weight (Mw) degradation of the PP solution was monitored by SDS-PAGE. A previous study has illustrated that whey protein is hydrolyzed into polypeptides at pH 2.0 under heating, some of which are assembled into amyloid fibrils [28]. PP gradually hydrolyzed during fibrillation, producing many peptides with small Mw, less than 10 kDa (Figure 1B). When heated for 8 h, bands with Mw between 15 and 50 kDa almost disappeared. When heated for more than 12 h, bands with Mw higher than 65 kDa almost disappeared. The bands at 70 and 94 kDa were actually attributed to the convicilin subunit and lipoxygenase, respectively. The band corresponding to lipoxygenase disappeared with only 2 h of heating, while the band corresponding to the convicilin subunit still was present after heating for 8 h. This was probably due to the higher thermal stability of convicilin with more disulfide bonds [29]. When heated for more than 24 h, bands with more than 10 kDa almost disappeared. This indicated that PP at pH 2.0 will undergo progressive hydrolysis during long-term heating. This is consistent with previous research results [15]. The results of the change in PP’ Mw demonstrate that the hydrolysis of PP into polypeptides, which can be assembled into amyloid fibrils, is a gradual process, requiring heating for a long time (24–48 h) with the temperature above the protein denaturation temperature at pH 2.0, which is far away from its isoelectric point.

#### 3.1.3. Atomic Force Microscopy (AFM)

Figure 1C shows the atomic force microscope image of PP fibrils as a function of heating time. The results show that PP in the original state is relatively uniform and spherical, and the morphology of PP changes significantly after heating for various durations. The morphology changes were not remarkable with 4 h of heating. After 12 h of heating, there were small fragments of protein that were hydrolyzed, some fragments of fiber that were assembled, and some slightly elongated protein fibrils. Compared with 12 h, the length of fibrils was significantly improved after heating for 24 h, indicating that a large number of fibrils were self-assembled during 12–24 h, which was the critical period of fibril growth, consistent with the ThT fluorescence results. Both wormlike and amyloid fibrils can be obviously observed on the AFM image. When heating for 36 h, compared with heating for 24 h, the length of some fibrils increased, while some fibrils broke. This was because when self-assembly was carried out, long-term heating could destroy the ordered structure of some fibrils, leading to fibril fracture.

#### 3.1.4. Free Sulfhydryl Content

Sulfhydryl groups can form intramolecular disulfide bonds to maintain the spatial conformation and can also combine with the sulfhydryl groups of other protein molecules to form intermolecular disulfide bonds, thus forming oligomers. Disulfide bond content and changes in the spatial structure of PP during the fibrillation process can be determined by measuring the content of the free sulfhydryl group in protein molecules. Figure 1D shows that the content of free SH in PP progressively decreased during fibrillation, indicating the formation of disulfide bonds with the free sulfhydryl group of PP. Meanwhile, more fibril-like structures were formed during fibrillation, and the spatial structure changed, resulting in some free SH groups being buried in the fibril structure [30]. Therefore, the content of the free sulfhydryl group in PP exhibited a decreasing trend during fibrillation. This indirectly illustrates the process of fibrillation. The free sulfhydryl content tended to be stable after 24 h fibrillation, indicating that mature PP fibrils were formed.

#### 3.1.5. Reducing Power

**Figure 2:**
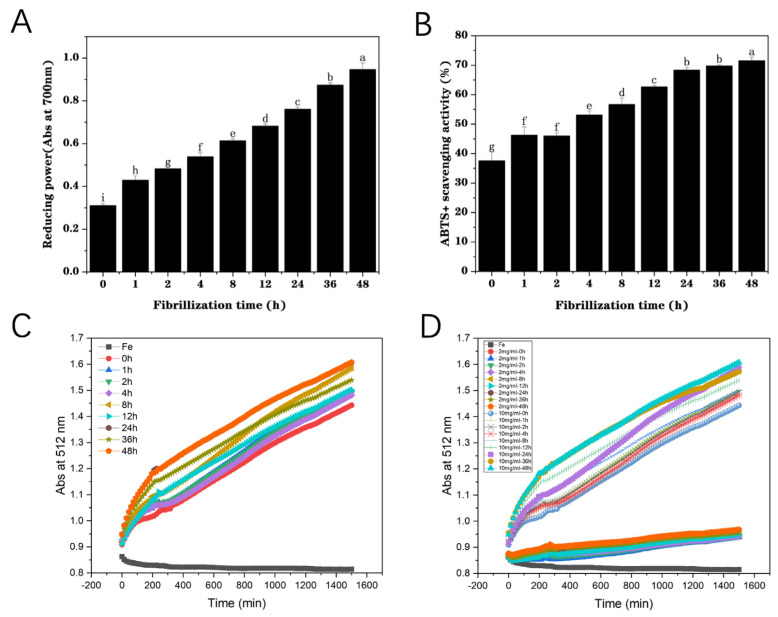
Reducing power of pea protein through heating at 85 °C for different times (0, 1, 2, 4, 8, 12, 24, 36, and 48 h) at pH 2.0 (**A**). Various letters (a–i) mean the significant difference (*p* < 0.05). ABTS ^+^ scavenging activity assay of pea protein heating at 85 °C for different times (0, 1, 2, 4, 8, 12, 24, 36, and 48 h) at pH 2.0 (**B**). Various letters (a–g) mean the significant difference (*p* < 0.05). The reducing effects of pea protein fibrils obtained at various heating times (0, 1, 2, 4, 8, 12, 24, 36, and 48 h) at 85 °C on iron (**C**). The reducing effects of pea protein fibrils with two various concentrations (2 g/L and 10 g/L) obtained at various heating times (0, 1, 2, 4, 8, 12, 24, 36, and 48 h) on iron (**D**). Pea protein fibrils and FeCl_3_ were analyzed individually as controls.

Food proteins possess a certain extent of antioxidant activity, even though less than that of small-molecule nutraceuticals such as carotenoids, polyphenols, and so on [31]. The reducing power of pea protein (0 h) was found to be approximately 0.31 (Figure 2A). With the increase in incubation time from 0 to 48 h, the reducing power of fibrils gradually enhanced from 0.31 to 0.92. This indicated that the reducing capacity of PP significantly improved during fibrillation. Increasing amounts of sulfhydryl groups and amino acid residues with reducing power were used to promote the improvement in their antioxidant properties in the process of assembling globular proteins into fibrils [32]. Moreover, the structural unit polypeptides of PP fibrils have a relatively high aspect ratio, facilitating the interaction between amino acid residues and metal ions, which is conducive to enhancing the reducibility [10]. Fibrillation treatment led to the exposure of the reducing groups of the proteins and improved their reducibility and antioxidant properties. Therefore, PP fibrils can be used as versatile carriers of bioactive substances, which can prevent further oxidation of nutrients.

#### 3.1.6. ABTS^+^ Scavenging Activity Assay

ABTS^+^ scavenging capacity can be used to detect the antioxidant activity of food proteins or peptides [33]. With the increase in heating time under acidic conditions, PP gradually hydrolyzed into small-molecule polypeptide fragments and self-assembled to form amyloid-like fibrils. The enhancement of fibrillation extent led to a progressive rise in the ABTS+ scavenging ability of PP (Figure 2B), which may be due to the formation of a high aspect ratio network structure of PP fibrils, which promoted the interaction between amino acid residues and free radicals. In addition, the destruction of the higher-order structure (secondary and tertiary) of the PP facilitated the exposure of amino acid groups to the water phase, which effectively removed free radicals, leading to an increase in the antioxidant activity of PP [34]. Fibrillation could markedly improve the ABTS ^+^ scavenging activity of PP from 37% to 71%, indicating that fibrils were better carriers and stabilizers of bioactive compounds, which was consistent with the results of reducing power detection.

### 3.2. Reducing Effect of Fibrillization on Iron Ions

The results of the antioxidant activity analysis (above) illustrated that the fibrillation degree of pea protein fibrils was positively correlated with its reducing power. As can be seen in Figure 2C, with the enhancement of PP fibrillation degree, the orange–red color of the Fe (II)-phenphenline complex progressively deepened, indicating an increase in the concentration of Fe (II) ions. This result demonstrated that the reducibility of PP fibrils was much higher than that of PP (native), and the higher the degree of fibrillation, the better the reducibility. In addition, the influences of PP fibril concentration on iron reduction capacity were also detected (Figure 2D). As expected, the PP fibril concentration played a critical role in its reducing power. There was a strong positive correlation between the PP fibril concentration and the reduction capacity, indicating that the higher the protein fibril concentration, the stronger the reduction capacity. These outcomes demonstrate that PP fibrils can effectively reduce Fe (III) to bioavailable Fe (II) and therefore is a great iron stabilizer and can be used as a relatively ideal iron carrier.

### 3.3. Characterization and Morphology of Fe-Fib PP

**Figure 3:**
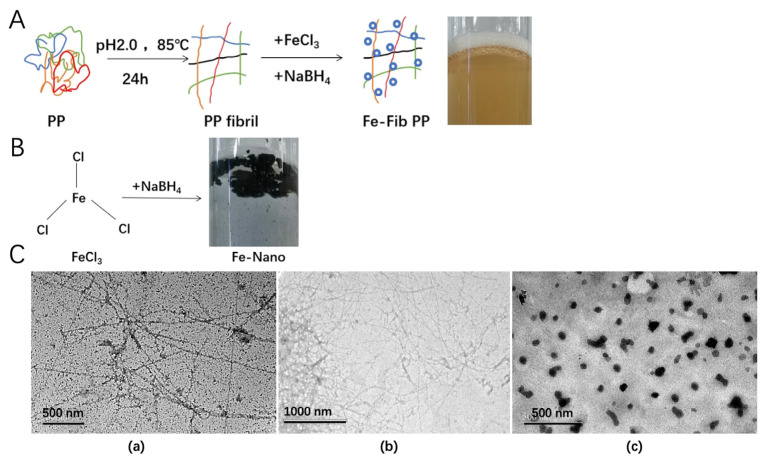
Schematic illustrations of the fabrication of pea protein fibrils and Fe-Fib PP nanocomposites (**A**). Schematic illustrations of Fe–Nano in the absence of pea protein (**B**). TEM images (**C**) of Fe-Fib PP (**a**), Fib PP (**b**), and Fe–Nano (**c**).

Iron nanoparticles are currently an important ingredient used as food iron fortification agents, exhibiting good bioavailability [35]. The addition of NaBH_4_ can reduce iron ions to zero-valent iron nanoparticles [36]. However, iron nanoparticles (Fe–Nano) dispersion was extremely unstable in the absence of stabilizers (like proteins) under reducing conditions and prone to precipitate rapidly into black iron clusters (Figure 3B), demonstrating that they could be easily oxidized and aggregated in solution. Interestingly, Fe-Fib PP dispersion was found to be transparent and stable, and no aggregates were observed during storage (Figure 3A). The morphology and size of PP fibrils formed at pH 2.0 with 24 h thermal treatment were similar to those of soy protein fibrils, having a highly flexible, linear shape with curled wormlike fibrils (Figure 3C) [27]. The results are consistent with the observation AFM and TEM. The process of fibrillation enhances the aspect ratio of PP and provides a spatial structure for iron nanoparticles to attach uniformly. However, it is very different with whey protein fibrils [37]. Iron nanoparticles can be clearly seen on the surface of the fibrils, and they are distributed evenly. This demonstrates that PP fibrils can effectively prevent iron nanoparticles from aggregation and precipitation and remarkably enhance their storage stability and possible bioavailability, illustrating that, compared to pure iron nanoparticles, PP fibrils are more suitable iron carriers to use as iron supplements in functional foods.

### 3.4. Stability of Iron–Pea Protein-Isolated Fibril (Fe-Fib PP) Nanocomposites and Iron Nanoparticles (Fe–Nano)

**Figure 4:**
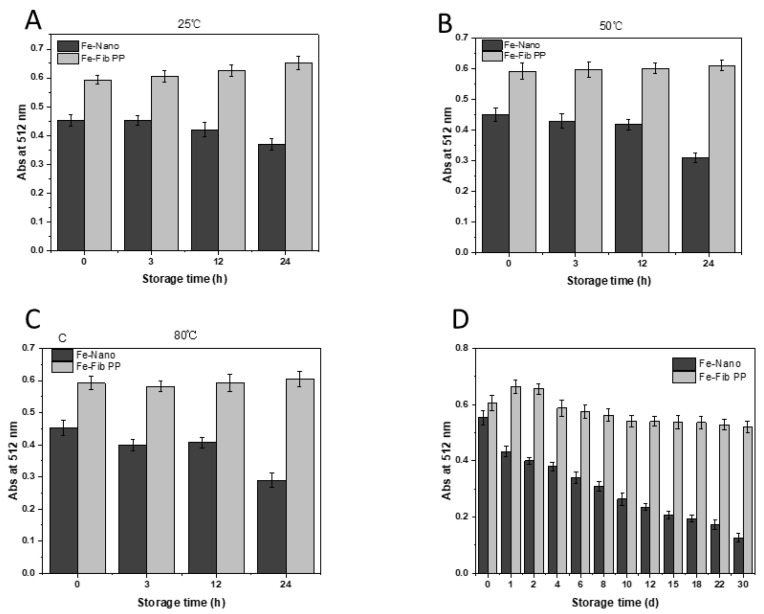
Influences of storage at various conditions on the stability of iron in Fe–Nano and Fe-Fib PP nanocomposites. Influences of storage temperatures 25 °C (**A**), 50 °C (**B**), and 80 °C (**C**) on the stability of iron in Fe–Nano and Fe-Fib PP nanocomposites (0, 3, 12, and 24 h). Influences of storage time (30 days) on the stability of iron in Fe–Nano and Fe-Fib PP nanocomposites at ambient temperature (**D**).

Increasing the content of Fe (II) and preventing further oxidation of Fe (II) are urgent problems to address. Heating treatment accelerates the oxidation of Fe (II), resulting in a decrease in Fe (II) content [38]. The heat stability of Fe-Fib PP nanocomposites and Fe–Nano solutions was assessed by testing the content of Fe (II) ions when stored at varied temperatures (25 °C, 50 °C, and 80 °C) for 24 h. The Fe (II) content of Fe-Fib PP was detected with 1,10-phenanthroline. As depicted in Figure 4, the Fe (II) content of freshly prepared Fe-Fib PP was much higher than that of Fe–Nano, signaling that Fe-Fib PP was more conducive to the absorption of iron by the human body. This was mainly attributed to the excellent reducing power of pea protein fibrils due to the high aspect ratio and the exposure of interior amino acid residues with strong antioxidant activities, which could pronouncedly promote the reduction of residual Fe (III) to Fe (II) ions. The content of Fe (II) in Fe–Nano stored at high temperature was significantly lower than that at room temperature for 24 h, indicating that Fe (II) oxidation was accelerated with the increase in temperature, and thermal treatment could accelerate the oxidation of iron, resulting in a lower content of Fe (II). The Fe (II) content did not change significantly when Fe-Fib PP was stored for 24 h at different temperatures, demonstrating that Fe-Fib PP could effectively protect iron from oxidation and improve the bioavailability of Fe (II).

Fe (II) can be easily oxidized to Fe (III) during storage, thus reducing the bioavailability of Fe (II). The stability and protective effect of Fe-Fib PP and Fe–Nano on Fe (II) were characterized by detecting the content of Fe (II) during 30 d storage at room temperature [39]. The absorbance of Fe-Fib PP exhibited a slight increase after 1 d storage (Figure 4), suggesting that the content of Fe (II) ion increased. However, a slight declining trend was found during the whole 30 d storage. However, no remarkable variations were observed between the content of Fe (II) of freshly prepared Fe-Fib PP and that after 30 d storage. Compared with Fe-Fib PP, the Fe (II) ion content of Fe–Nano was less, and the content of Fe (II) in Fe–Nano was dramatically decreased within 30 d storage. The outcomes obviously demonstrated that NaBH_4_ could rapidly reduce some Fe (III) ions, but the reducing agent pea protein fibrils could markedly promote the reduction of residual Fe (III) to Fe (II) ions and keep the amount of Fe (II) stable in a long time.

### 3.5. XPS

**Figure 5:**
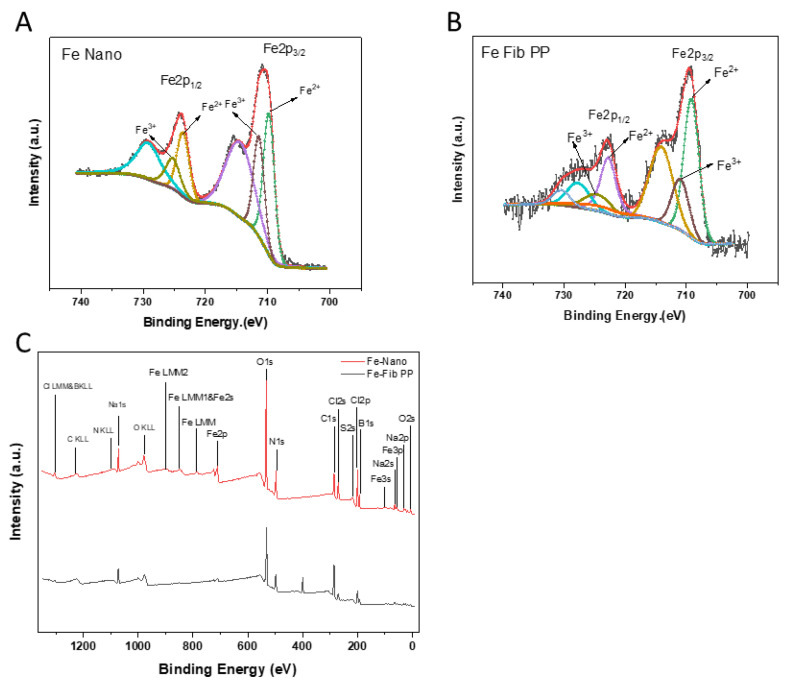
Fe2p3/2 and Fe2p1/2 spectra of Fe–Nano (**A**) and Fe-Fib PP (**B**). All samples were freeze-dried before XPS characterization. XPS survey spectra (binding energy (eV) vs. intensity (a.u.) of Fe-Fib PP and Fe–Nano (**C**).

Food-derived iron fortification is considered to be an effective method to solve iron deficiency anemia. However, the chemical form of iron, as well as the amount of iron, can limit the bioavailability of iron [40]. The valence state and content of iron in the samples were detected with XPS. The high-resolution ferro-spectra of Fe2p3/2 and Fe2p1/2 and XPS images are shown in Figure 5A–C. In general, C, O, Fe, and N signals were obviously observed, and a small number of Na, B, and Cl signals were also detected, which may occur during sample preparation. High-resolution ferro-spectra of Fe2p3/2 and Fe2p1/2 of Fe–Nano and Fe-Fib PP and their satellite peaks were obtained. In the spectrum of Fe-Fib PP, the signals at 709.1 eV and 722.7eV accounted for 70.75% of the total peak area (Table 1). The signals of its vibrating satellite at 714.09 eV and 727.7 eV were classified as Fe (II) oxides [41]. Additionally, the signals at 711 eV and 724.6 eV accounted for 29.25% of the total peak area, and the signal of their vibrating satellite at 724.1 eV and 730.48 eV were classified as Fe (III) oxides [41]. For Fe–Nano, as detected by XPS, Fe (II) and Fe (III) accounted for 56.05% and 43.95% of the total area, respectively. It can be clearly seen that Fe (II) content in Fe-Fib PP was remarkably higher than that of Fe–Nano, suggesting Fe-Fib PP is more conducive to human absorption [7]. The results demonstrate that Fe-Fib PP exhibits great potential as an effective iron-fortifying agent.

### 3.6. Bioaccessibility

**Figure 6:**
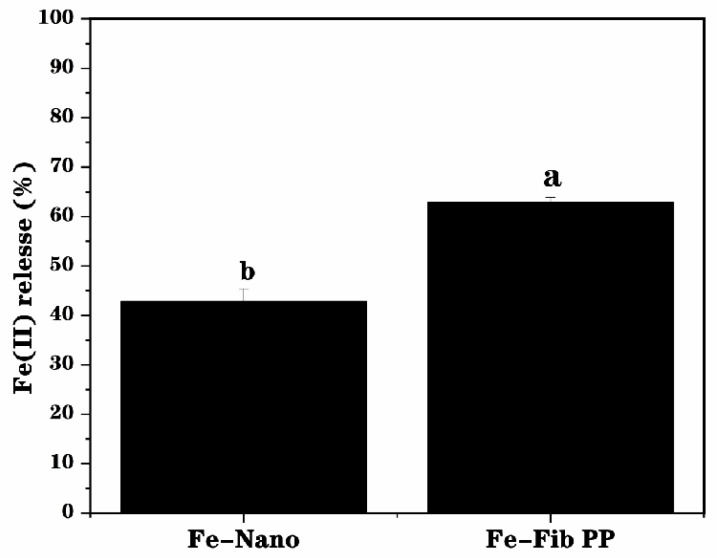
The Fe (II) release amount (%) from Fe-Fib PP and Fe–Nano during *in vitro* simulated gastrointestinal digestion. Various letters (a,b) mean the significant difference (*p* < 0.05).

The bioaccessibility of iron nanoparticles (Fe–Nano) and Fe-Fib PP was tested by simulating gastrointestinal digestion. After digestion, compared with Fe–Nano (42.7%), Fe (II) release of Fe-Fib PP was found to be 62.8% (Figure 6), demonstrating the bioaccessibility of Fe-Fib PP was significantly improved, which may be due to the fact that Fe–Nano was more likely to accumulate during digestion, being not conducive to the release of Fe (II). Furthermore, compared with Fe-Fib PP, Fe (II) in (Fe–Nano) can be easily oxidized during digestion, resulting in a decrease in Fe (II) content. By contrast, PP fibrils made the iron nanoparticles more dispersed and stable due to the high aspect ratio and spatial structure, as well as strong antioxidant activity, and thus they were more conducive to the release of Fe (II). Iron nanoparticles attached to the PP fibrils can be clearly seen through TEM images (Figure 3). Fe–Nano, however, underwent a certain degree of aggregation. This was consistent with the previous results of reducing power and ABTS^+^ scavenging activity assay. Therefore, compared with Fe–Nano, Fe-Fib PP is a good, cost-effective, and highly absorbent iron nutrient enhancer.

## 4. Conclusions

In this work, amyloid fibrils were prepared with typical, novel, and useful plant protein–pea proteins, and the antioxidant activity and iron reduction in pea protein fibrillar aggregates and Fe-Fib PP nanocomposites were comprehensively investigated. The reduction effect is highly dependent on the degree of fibrillation and fibril concentration. A new stable Fe-Fib PP nanocomposite, which can be used as an iron fortificant, was obtained using the antioxidant properties of pea protein fibrils. Compared with iron nanoparticle solution without PP fibrils (Fe–Nano), Fe-Fib PP nanocomposites showed high dispersibility, stability, and bioaccessibility. In addition, under different temperatures and long-term storage conditions, Fe-Fib PP exhibited better stability and could prevent Fe (II) from oxidation. The retention content of Fe (II) was much higher in Fe-Fib PP during storage. Since Fe (II) is more easily absorbed and utilized by the human body, Fe-Fib PP can be expected to possess better bioaccessibility. Overall, PP fibrils with strong antioxidant activities can be used as iron carriers in food systems. The fabricated Fe-Fib PP nanocomposite is a great food iron fortifying agent for the treatment and prevention of IDA, which could provide some useful information for the research of food iron fortification.

## Figures and Tables

**Table 1:** Mean value (%) of peak area of Fe (II) and Fe (III) of dried compounds (Fe–Nano and Fe-Fib PP) from XPS measurements.

Peak Area (%)	Fe–Nano	Fe-Fib PP
Fe (II)	56.05 ± 0.50%	70.75 ± 0.65%
Fe (III)	43.95 ± 0.75%	29.25 ± 0.30%

## Data Availability

The original contributions presented in this study are included in the article. Further inquiries can be directed to the corresponding authors.
